# Long-term data reveal unimodal responses of ground beetle abundance to precipitation and land use but no changes in taxonomic and functional diversity

**DOI:** 10.1038/s41598-021-96910-7

**Published:** 2021-09-01

**Authors:** Petr Zajicek, Ellen A. R. Welti, Nathan J. Baker, Kathrin Januschke, Oliver Brauner, Peter Haase

**Affiliations:** 1 Department of River Ecology and Conservation, Senckenberg Research Institute and Natural History Museum Frankfurt, Gelnhausen, Germany; 2 Department of Aquatic Ecology, University of Duisburg-Essen, Essen, Germany; 3 Office for Zoology, Vegetation and Conservation (Büro für Zoologie, Vegetation und Naturschutz), Eberswalde, Germany; 4 Faculty of Biology, University of Duisburg-Essen, Essen, Germany

**Keywords:** Ecology, Biodiversity, Community ecology

## Abstract

While much of global biodiversity is undoubtedly under threat, the responses of ecological communities to changing climate, land use intensification, and long-term changes in both taxonomic and functional diversity over time, has still not been fully explored for many taxonomic groups, especially invertebrates. We compiled time series of ground beetles covering the past two decades from 40 sites located in five regions across Germany. We calculated site-based trends for 21 community metrics representing taxonomic and functional diversity of ground beetles, activity density (a proxy for abundance), and activity densities of functional groups. We assessed both overall and regional temporal trends and the influence of the global change drivers of temperature, precipitation, and land use on ground beetle communities. While we did not detect overall temporal changes in ground beetle taxonomic and functional diversity, taxonomic turnover changed within two regions, illustrating that community change at the local scale does not always correspond to patterns at broader spatial scales. Additionally, ground beetle activity density had a unimodal response to both annual precipitation and land use. Limited temporal change in ground beetle communities may indicate a shifting baseline, where community degradation was reached prior to the start of our observation in 1999. In addition, nonlinear responses of animal communities to environmental change present a challenge when quantifying temporal trends.

## Introduction

The world is run by the little things^1^, with insects being incommensurately under-described, yet comprising over 1 million of the 1.7 million named living species on Earth^2,3^. However, disproportionately few long-term ecological monitoring programs have targeted insects, and large knowledge gaps remain for many insect groups regarding their temporal trends and potential drivers. For example, BioTIME, a database of published biodiversity time series from 362 studies, contains only 22 studies (6%) primarily targeting insects^4^.

Long-term monitoring of ecological communities is critical for understanding the dynamics of community change^4–6^. Although recent research suggests many terrestrial insect taxa are declining^7–11^, most studies have only examined changes in selected community characteristics such as abundance or biomass^7,8,12^, or a few facets of taxonomic diversity^13–15^ such as taxonomic richness or species turnover^12,16^. More recently, studies have begun to incorporate trait-based approaches to investigate functional diversity^17^, an approach that offers valuable insights to unravel community change and its consequences for ecosystem functioning^18^. One of the most widely used methods for estimating functional diversity is a multidimensional trait-based approach which uses species-specific trait data^19^. Species traits express a measurable property of an organism^18^ such as particular requirements on habitats (e.g., habitat specialists) or feeding habits (e.g., predators) and are therefore directly linked to ecosystem function^20–22^. However, long-term research assessing temporal community trends of both taxonomic and functional diversity and the potential drivers thereof remain scarce.

Climate change and land use intensification are two of the overarching anthropogenic drivers of taxonomic and functional diversity^23–26^ and are the leading hypothesized causes of insect declines^9,27–30^. Climate change can directly affect insects when novel climate conditions continuously exceed insects’ historical tolerance limits^31^ and can favor particular taxa such as those with elevated dispersal abilities^32^. While climate change acts on broader scales, land use affects communities at local scales^33^. Land use can directly reduce habitat quantity and quality, reducing insect abundance and altering community composition^34^. Ground beetles (Carabidae) are considered relevant bioindicators for conservation and habitat restoration^35,36^. Ground beetles are common, taxonomically well described^37^ and highly diverse, with more than 3000 species in the Western Palearctic region^38,39^. Additionally, ground beetles are sensitive to both changes in climate^40–42^ and land use intensification^43–46^.

Recent long-term studies of western European ground beetle communities have reported high variation in temporal patterns. In the Netherlands, total numbers of individuals declined within one region from 1985 to 2016^47^. In the UK, species richness declined while Shannon’s diversity remained stable at eleven sites between 1994 and 2008^48^. Declines in the numbers of species over 50–100 years in Belgium, Denmark, and the Netherlands were related to an increase in densities of generalists^49^. In Northern Germany, species richness, phylogenetic diversity and species which always or sometimes hibernate as imagines declined whereas biomass remained stable at one site between 1994 and 2017^50^. At two Scottish sites between 1994 and 2011, the abundance of generalist species increased while the overall abundance, species richness and diversity remained stable^40^. These studies generally only include a few sites or regions but do highlight the importance of assessing both taxonomic and functional diversity as each can vary independently of the other^22^.

Ground beetles vary widely in environmental adaptations such as thermal and moisture tolerances^51^, with changing temperatures and precipitation favoring particular species at the expense of others^40–42,52^. Macro- and micro-climate conditions affect ground beetle communities^42^ both directly and through effects on habitat structure or biotic interactions; at the macroscale, temperature can decrease taxonomic diversity both within and between forested patches, while locally, increased moisture due to canopy cover can be favorable for habitat generalists, non-forest and open-habitat species^42^. Higher temperatures may increase adult ground beetle overwintering survival or activity levels, resulting in higher abundances^53,54^. Alternatively, a combination of hot and dry temperatures can desiccate beetle larvae^50^ and reduce their abundance. In habitats adjacent rivers, high precipitation can increase detritus deposition on shorelines, providing additional resources and habitat to ground beetles^55^. Furthermore, precipitation is essential to maintain riparian habitats, and flooding as a result of high precipitation can promote specialist species and those with high dispersal abilities (e.g., hygrophilous and winged beetles)^56,57^, while the absence of flooding events may favor generalist species and those with reduced dispersal abilities^57^.

To comprehensively unravel ground beetle community trends and their drivers, we simultaneously examined several characteristics of taxonomic diversity (including temporal turnover), functional diversity, activity density (a proxy for abundance) of all ground beetles, and activity densities of functional groups at 40 sites in five regions in Germany over the last two decades. We aimed to determine whether temporal trends in ground beetle communities are driven by climate change (temperature and precipitation) and land use intensity, and asked how trends in taxonomic diversity are reflected by trends in functional diversity. We hypothesized that (1) consistent with recent long-term analyses from Germany and neighboring countries^47,50,58^, ground beetle taxonomic diversity will decrease over time and functional diversity will decrease as specialists decline, (2) increasing temperatures and alterations in precipitation will increase temporal species turnover as beetles with differing thermal and moisture preferences enter and exit local communities^51^, and (3) land use intensification will reduce taxonomic and functional diversity and increase the activity density of habitat generalists.

## Methods

### Sites

**Figure 1:**
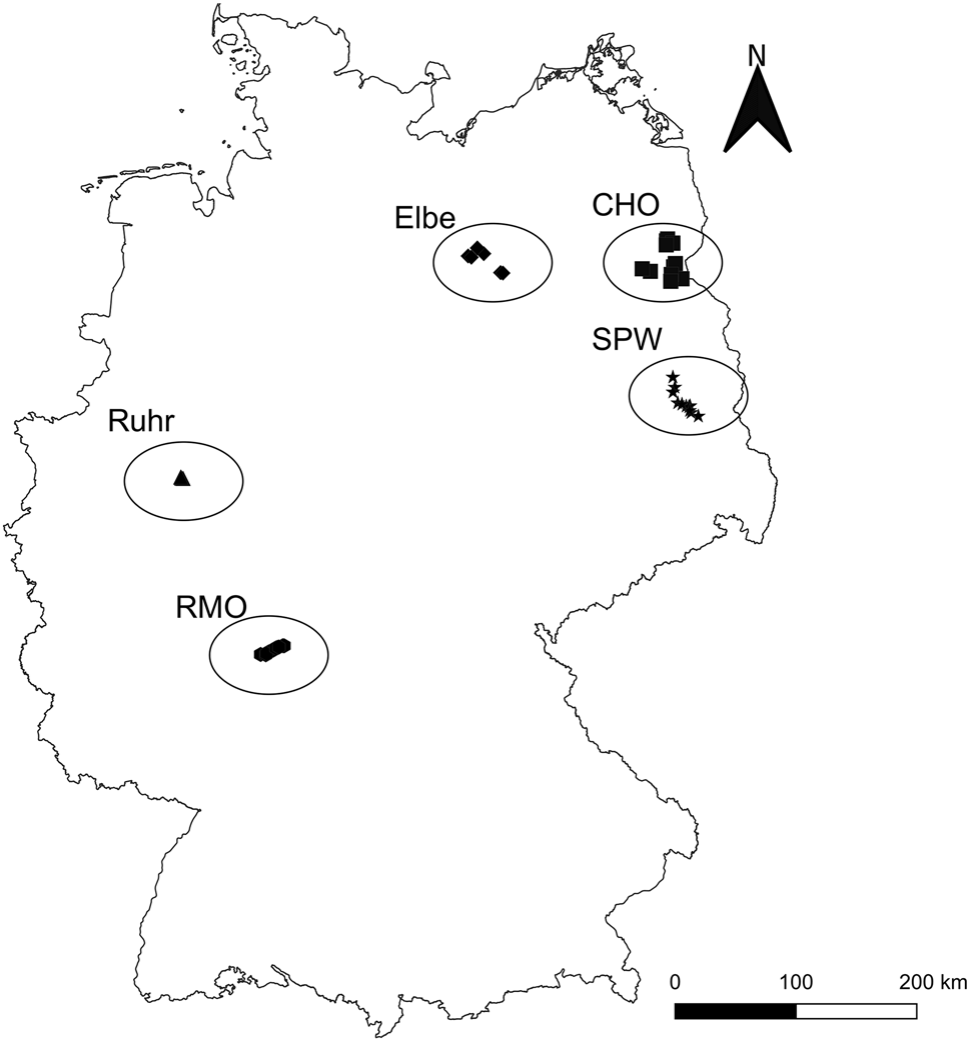
Location of the 40 sampling sites within five regions in Germany. Within regions, sites have identical symbols; ovals around the sites represent regions. Region labels are described in Table 1.

Overall, ground beetle sampling was conducted at 40 sites within five regions across Germany between 1999 and 2019 (Fig. 1). The five sampling regions were: the Rhine-Main-Observatory (https://deims.org/9f9ba137-342d-4813-ae58-a60911c3abc1), a Long-Term Ecosystem Research (LTER) site along the Kinzig River (n = 10 sites, RMO region); the Ruhr River (n = 4 sites, Ruhr region); the ‘Flusslandschaft Elbe Brandenburg’ biosphere reserve along the Elbe River (n = 6 sites, Elbe region); the ‘Schorfheide-Chorin’ biosphere reserve and LTER site (https://deims.org/94c53dd1-acad-4ad8-a73b-62669ec7af2a, n = 9 sites, CHO region); and the ‘Spreewald’ biosphere reserve (n = 11, SPW region). Sampling sites covered heterogeneous environments representing 11 habitat types (as described by the experts who conducted the sampling: RMO = meadow, urban, forest; Ruhr = river floodplain; Elbe = grassland within floodplain, grassland disconnected from floodplain; CHO = arable land, marsh, marsh meadow; SPW: meadow, arable marsh, rewetted marsh, arable land).

### Ground beetle sampling

Ground beetle sampling followed standard procedures using either unbaited pitfall traps (either six or eight traps per site)^59,60^ or both unbaited pitfall traps and hand collections^61,62^. In the Ruhr region, pitfall trapping was supplemented with a total of 204 hand captures following standardized procedures^61,62^. Beetles collected by hand captures were only used in analyses based on within-site comparisons and were excluded from analyses of activity density based on across-site comparisons. Sampling methodology was identical across years within each site but varied between regions. All sites (1) had an observation period (= study length) of at least ten years except one site with nine years (averaging 13.9 yrs ± 0.59 SE), (2) had at least four years of sampling within the observation period (averaging 7 yrs ± 0.33 SE), and (3) were sampled in the same season within the time series^63^. To maintain a minimum sample size of at least four sites per region, we included one site (in the Ruhr region) with an observation period covering nine years (and eight sampling years). All regions were sampled between May and July except the RMO region, which was sampled between August and September (accounted for in statistics). Captured individuals were stored in ethanol^64,65^ and identified to species^66^.

### Community metrics

For each sampling event (per site and year), we calculated 21 taxonomic and functional community metrics (Table 1) representing taxonomic diversity, taxonomic turnover, activity density, activity densities of functional groups and functional diversity.

**Table 1:** Community metrics, regions and their abbreviations evaluated in this manuscript.

Community metrics		
1	SPR	Species richness
1	SHA	Shannon Index
1	SIM	Simpson Index
1	EVE	Evenness (Pielous' J)
1	Evar	Evar Evenness
2	TUR	Turnover
2	APP	Species appearance
2	DIS	Species disappearance
2	SERa	Species exchange rate
3	ABU	Activity density (abundance)
4	SPE	Activity density of habitat specialists
4	GEN	Activity density of habitat generalists
4	PRE	Activity density of predators
4	IMA	Activity density of hibernating beetles as imagines
4	LAR	Activity density of hibernating beetles as larvae
4	WIN	Activity density of winged beetles
4	DIM	Activity density of dimorphic beetles
5	FRic	Functional richness
5	FEve	Functional evenness
5	FDiv	Functional divergence
5	FDis	Functional dispersion

Regions		
RMO		Rhine-Main-Observatory (LTER-site)
Ruhr		Ruhr River
Elbe		Biosphere reserve Flusslandschaft Elbe Brandenburg
CHO		Biosphere reserve Schorfheide-Chorin (LTER-site)
SPW		Biosphere reserve Spreewald

1 = taxonomic diversity, 2 = taxonomic turnover, 3 = activity density 4 = functional traits, 5 = functional diversity.

#### Taxonomic diversity and activity density

To assess taxonomic diversity, we calculated five community metrics: species richness, Shannon’s diversity^67,68^, Simpson’s diversity^69^, evenness (Pielou’s J)^70^, and ‘Evar’ evenness^71^. We opted to assess Evar in addition to Pielou’s J as Evar is equally sensitive to rare and abundant species and therefore considered more appropriate than Pielou’s J^71^, which on the other hand is more commonly used in other studies and therefore offers better comparability. To assess taxonomic turnover, we calculated four community metrics: turnover (number of species ‘appearances’ and ‘disappearances’ divided by the total number of species) and its components ‘species appearances’ (immigrations) and ‘species disappearances’ (extinctions) and its complementary species exchange rate (SERa) according to the formula provided in Hillebrand et al.^72^. SERa takes into account species proportional abundances and is therefore considered a more robust measure to detect compositional change through time compared to turnover^72^. The number of ground beetles captured with pitfall traps are considered measures of ‘activity density’ rather than true abundance as they reflect both the density of individuals and their level of locomotion facilitating their capture^51^.

#### Functional traits

We considered four trait groups representing seven functional traits^73^, hereafter functional groups: habitat preference (specialists and generalists; species inhabiting only one habitat class were considered specialists, all others generalists), flying ability (winged and dimorphic), hibernation stage (imago and larva), and feeding habits (predators and herbivores, only predators common enough to examine individual trends in activity densities). The selected traits address physiological and behavioral aspects of a species survival, particularly regarding land-use intensity (habitat preference)^42^, resource acquisition (feeding habits)^55^, dispersal and dissemination (flying ability)^56,57^, and resistance to harsh conditions (hibernation stage)^54^. Functional traits were retrieved from www.carabids.org—an online database of ground beetle species traits^37^ (accessed 29.07.2020).

#### Functional diversity

We calculated four distance-based functional diversity metrics based on Gower dissimilarity according to Villéger et al.^74^ and Laliberté and Legendre^75^: functional richness (the niche space occupied by all species of the community^76^), functional evenness (functional group distribution of abundances across the niche space^77^), functional dispersion (the average distance of individual species to the functional group centroids of all species^74^), and functional divergence (a measure of how spread or clumped species are within the niche space, weighted by the relative abundance^76,78^). Prior to analyses, species-specific traits (functional groups) were fuzzy-coded between 0 and 3 following Chevenet et al.^79^. Dimensionality reduction was required during the calculation of functional richness; the final quality of the multidimensional trait-space was 0.61.

### Climate data

We retrieved daily temperature and precipitation data from the gridded observational European dataset (E-OBS Temperature and Precipitation data set)^80,81^ with a spatial resolution of 0.1 degrees. We then calculated the mean annual daily temperature and annual cumulative precipitation of the 12 months preceding each sampling event to account for climatic conditions across the full range of ontogeny^82^.

### Land use data

We extracted land use data around each sampling site from the CORINE land cover dataset^83^, with the highest resolution (5 ha) available from the year 2012. CORINE land cover data provide spatial coverage of land use types and were available for the years 2012 and 2018. We selected the year 2012 as it represents the mean of the timespan covered by our time series. Land use types were assumed to be consistent during the observation period as all sites are situated in biosphere reserves or along river floodplains. As an indicator for land use intensity, we calculated the land use index (LUI)^84^ based on the coverage of land use categories within a radius of 500 m (LUI_500), 1000 m (LUI_1000) and 2000 m (LUI_2000) around ground beetle sampling sites.

$${\text{LUI}} = \% {\text{pasture}} + {\text{2 x }}\% {\text{arable}}\;{\text{land}} + {\text{4 x }}\% {\text{urban area}}$$

The three different buffers of land use intensities were all highly correlated (R^2^ = 0.88, *p* < 0.001). Hence, we only included LUI_1000 as site-specific estimates of LUI.

### Data analysis

#### Standardization of community metrics

To avoid bias in richness metrics (taxonomic and functional diversity) due to variable exposure times of pitfall traps within sites over time, we used rarefied indices. All richness-related community metrics were rarified within a given site to the year with the shortest exposure time^85,86^. Rarefied community metrics are only used in analyses based on within-site comparisons. We refrained from applying rarefaction across sites to prevent rarefaction-induced bias of richness-related community metrics at sites with shorter exposure times (i.e., substantially reducing exposure time and thus removal of rarely captured species) and because the shape of rarefaction curves may vary across sites.

Activity density was standardized by dividing by the total exposure time of pitfall traps per site and year (units are the number of captured beetles per day) and used in analyses of across-site comparisons. Analyses based on within-sites comparisons also include hand captures (Ruhr region only) as those had a standardized (equal) sampling effort in each sampled year within the sites.

#### Statistical approach

We used two approaches to examine climate and land-use effects on ground beetle communities. The first approach focuses on temporal trends and their drivers and is based on within-site comparisons. Approach one is independent of local scale environmental heterogeneities between sites. The second approach takes advantage of temporal variation in community metrics and climate variables (of each sampled year at each site) in addition to spatial variation. Approach two examines the effects of climate and land use on ground beetle communities, but it is limited to changes in activity densities, which were comparable across sites. We summarize both statistical approaches briefly below and provide more details in the supplementary materials.

Approach one uses a meta-analytical approach based on Mann Kendall trend tests of time series within each site. It accounts for both temporal and spatial autocorrelation and tests for (1) overall and regional temporal trends in all ground beetle community metrics, (2) overall trends in temperature and precipitation, and (3) the effects of trends in climate variables and site-specific LUI’s on the overall trends in community metrics. Following Maire et al.^87^, we report the model coefficients from meta-analytical mixed effects models as trend mean effect sizes (TMES) and standard errors (SE). Observation period was additionally included in models to account for time series length. The major advantage of this approach is the ability to assess overall and regional trends based on heterogeneous site-specific time series (covering heterogeneous habitat types) for all community metrics.

With the second approach, we investigate whether site/year-specific variation in climate and site-specific variation in land use intensity explain variation in ground beetle activity densities. This approach tests for relationships between the drivers of site/year-specific temperature, precipitation, site-specific LUI’s and the responses of activity density, functional groups, and the dominance of functional groups (using axes of a Principal Component Analysis [PCA] to eliminate multicollinearity, appendix Fig. A1 & Table A1) using generalized least squares regression (gls) models. The major advantage of this approach is the ability to incorporate both temporal and spatial variation in predictors and responses. However, this approach could only be conducted for community metrics based on activity density as only activity density could be realistically standardized across sites.

#### Statistical software

All analyses were conducted in R 3.6.1^88^. Species richness, Shannon and Simpson diversity indices, evenness (Pielou’s J), turnover, species appearances and disappearances, species rarefaction, and PCA were calculated using package vegan, version 2.5-5^85^. The Evar metric was calculated using package microbiome, version 1.8.0^89^. Functional diversity metrics were calculated using the FD package, version 1.0-12^90^. Package metafor, version 2.4-0^91^ was used for meta-analyses and package glmulti, version 1.0.8 for multi model selection and inference^80^. Generalized least squares models were calculated using the package nlme, version 3.1-140^92^.

## Results

A total of 65,874 ground beetles from 194 species were captured in 46,566 days of pitfall trapping and 204 hand captures. Rarefaction was based on a total exposure time of 40,916 pitfall trapping days and hand captures, reducing the initial dataset to estimates of 59,548 ground beetles from 190 species. Analyses of spatio-temporal variation in community metrics based on activity density included 61,826 ground beetles from 184 species from pitfall traps only.

### Climate trends and land use gradient

Trends in mean temperature and cumulative precipitation over the 12 months prior to sampling indicated an increase in temperature (TMES ± SE: 11.07 ± 4.61; *P* = 0.016) and a marginal increase in precipitation (4.46 ± 2.40; *P* = 0.063) across all sites during the observation period (Appendix Fig. A2). Across all 40 sites and sampling years, annual mean temperatures varied from 7.8 to 12.6 °C and annual cumulative precipitation ranged from 424.5 to 878.4 mm (Appendix Fig. A3). The index of land use intensities (LUI) at buffers of 1000 m around sites averaged 144 ± 88 (mean ± SD, range: 10–375, Appendix Fig. A4).

### Temporal trends in community metrics and their drivers

**Figure 2:**
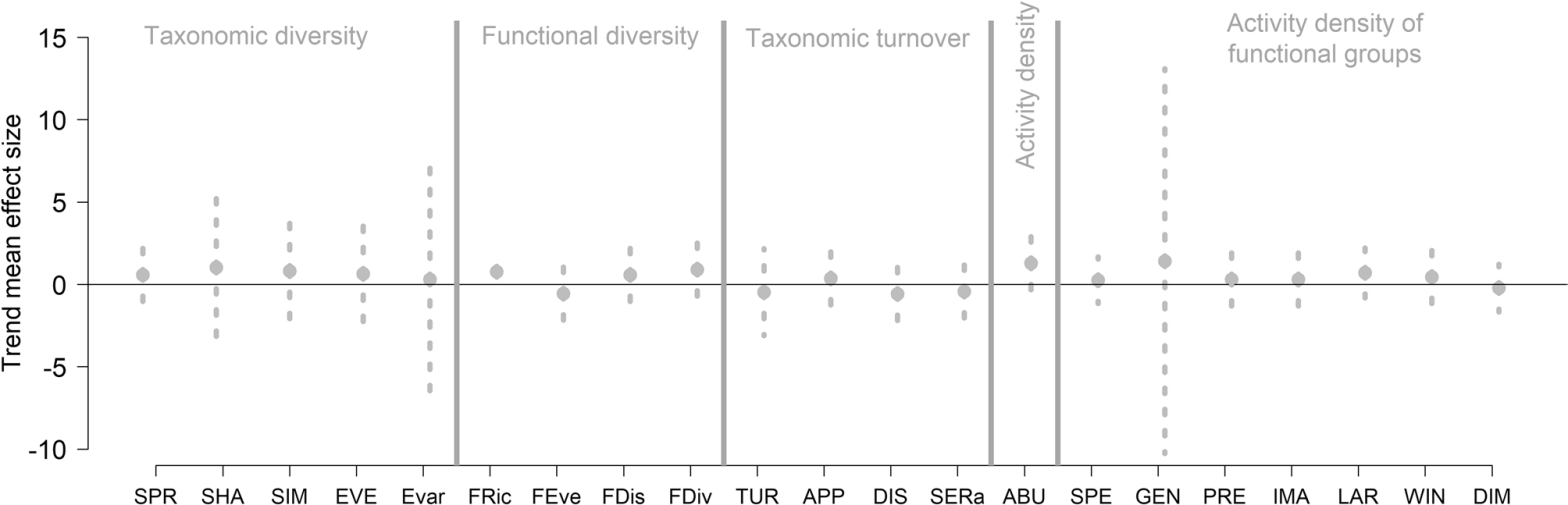
Overall trends in 21 community metrics representing changes in the taxonomic and functional composition of ground beetle communities over the last two decades at 40 sites in Germany. Grey dashed lines show 95% confidence intervals. Abbreviated community metrics on the y-axis are explained in Table 1.

On average across the 40 sites, none of the 21 assessed community metrics changed significantly over time (Fig. 2). Within regions, APP decreased (*p* = 0.007) whereas DIS increased (*p* < 0.001) in the CHO region and SERa increased (*p* = 0.002) in the SPW region (Appendix Fig. A5). No significant trends were evident in any of the other community metrics and the five regions (Appendix Figs. A5, A6, A7 & A8).

Site-based trends in SERa increased with trends in temperature (*p* = 0.032) and declined with site-specific LUIs (*p* = 0.002, Table 2). Site-based trends in SIM (*p* = 0.049), EVE (0.031) and FDis (0.017) declined, whereas SPE increased (*p* = 0.030, Table 2) with trends in precipitation. Evar declined with observation period (*p* = 0.010, Table 2).

**Table 2:** Drivers with a significant influence on the overall trends in community metrics.

Community metric	Predictors			
	Temperature	Precipitation	LUI_1000	Observation period
SERa	↗ **2.27**	↗ 0.82	↘ − **2.61**	
SIM		↘ − **1.97**	↗ 0.72	↘ − 0.74
EVE	↗ 0.36	↘ − **2.15**	↘ − 1.08	↘ − 1.23
Evar		↘ − 1.71	↘ − 0.66	↘ − **2.95**
SPE		↗ **2.29**	↘ − 0.97	↗ 0.93
FDis	↗ 1.42	↘ − **2.56**	↗ 0.78	

Arrows indicate the direction of effect sizes (z values) of influential drivers identified in the model selection and multi-model inference procedures. Bold font indicates significant drivers and their effect sizes. Abbreviations of community metrics are explained in Table 1.

### Drivers of spatio-temporal variation in activity density and functional groups

**Figure 3:**
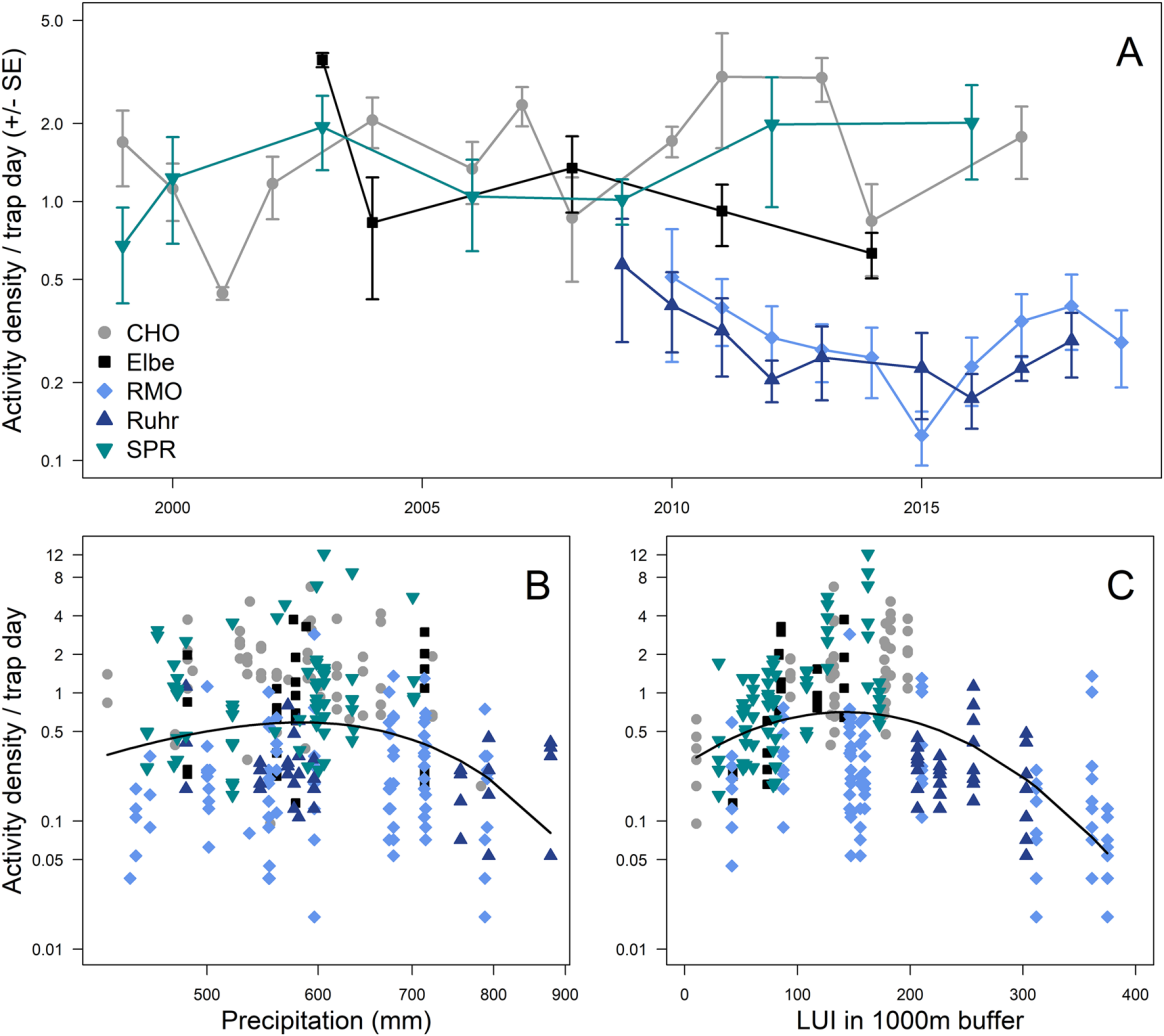
Spatio-temporal variation of activity densities across the five regions (**A**) and activity density responses to significant drivers from the overall model (Table 3): Precipitation (**B**) and land use intensity (LUI) around 1000 m of sampling sites (**C**). Error bars in Panel A represent one standard error. Points in Panels B and C represent activity densities within each site and year.

Activity densities from pitfalls were higher at the three northeastern regions (CHO, Elbe, SPW) than at the two southwestern regions (RMO, Ruhr; Fig. 3A). Functional group activity densities tended to parallel total activity densities, with the notable exception of much higher captures of predators in the Ruhr region (Appendix Fig. A9). Total activity density had a unimodal response to both precipitation (peaking at around 600 mm; Table 3 & Fig. 3B) and land use intensity (peaking at around LUI ~ 160 Table 3, Fig. 3C). All seven functional groups showed similar unimodal responses to LUIs (Appendix Table A2 & Fig. A10).

**Table 3:** Spatio-temporal drivers of overall ground beetle activity density.

	Est	SE	t-value	*P*
Intercept	0.5677	1.392	0.408	0.684
Temperature	− 0.486	0.608	− 0.8	0.425
Precipitation	1.2867	0.703	1.831	0.068
Precipitation, 2nd poly	− **2.078**	0.812	− 2.56	0.011
LUI_1000m	− **5.004**	1.642	− 3.05	0.003
LUI_1000m, 2nd poly	− **10.86**	1.579	− 6.87	< 0.001

The generalized least squares model included an autoregression term to account for temporal autocorrelation, a grouping factor of site nested within region to account for repeated sampling within sites. Estimates are shown in bold when significant. Second order polynomial terms are denoted as “2nd poly”.

The activity densities of three functional groups were driven by annual precipitation (Appendix Table A2, Fig. A11). Activity densities of generalists and winged beetles were highest at ~ 600 mm of annual precipitation and then declined at higher precipitation rates. Predators increased linearly with precipitation. Three functional groups: dimorphic beetles, larval-, and imago-hibernators had significant second order polynomial responses to temperature (Appendix Table A2E,F,G), though the fitted relationship was more indicative of linear declines in activity densities with temperature (Appendix Fig. A11D,E,F).

Ground beetle functional group dominance varied with land use intensity and annual precipitation. The first principal component (PC1) of the functional dominance PCA represents communities with high proportions of dimorphic beetles and low proportions of specialists, winged beetles, and imago-hibernators (Appendix, Table A1). PC1 had a u-shaped response to land use intensity, indicating specialists, winged beetles and imago-hibernators drove the pattern of increasing activity densities at intermediate land use intensities (Appendix Fig. A12). The second component (PC2) represents communities with high proportions of larval-hibernators and low proportions of predators. PC2 was negatively correlated with precipitation, indicating predators had increased dominance and larval-hibernators were proportionally less common with increasing annual precipitation (Appendix Fig. A12).

## Discussion

We did not detect any net directional temporal changes in ground beetle communities across the 40 sites over the last two decades in Germany. However, across all sites and sampling years, ground beetle activity densities peaked at intermediate annual precipitation and land use intensity, a result supported by other studies reporting compositional shifts across land-use gradients^45,46,93–95^. The unimodal response of activity densities to precipitation was most pronounced for habitat generalists and winged beetles, which may be indicative of better dispersion abilities of these groups to more suitable habitats^49^ following exceeded tolerance limits to precipitation^31^. Increases in activity density and functional dominance of predators with precipitation may be a consequence of rainfall-driven increases in habitat volumes resulting in extended food chain length^96^.

In contrast to our expectations of increased taxonomic and functional diversity (Hypothesis 1), we detected no temporal trends in the 21 analyzed community metrics representing taxonomic and functional diversity, taxonomic turnover, activity density, and activity densities of functional groups. However, we cannot reject our prediction that trends in functional diversity paralleled trends in taxonomic diversity as both taxonomic and functional diversity were fairly invariant over time. Our results are in line with other large-scale biodiversity assessments of various taxonomic groups reporting few long-term net changes in taxonomic diversity metrics^12,16,72,97^. These studies have generally reported overall increases in temporal turnover, whereas in our study, turnover changes were restricted to two regions (CHO and SPW, both in northeast Germany). Our results also contrast several local scale studies from the UK, Northern Germany and the Netherlands reporting declines in ground beetle taxonomic diversity and abundance^47,48,50^, and to recently reported declines in activity density and species richness in ground beetles at a single German site^98^. However, these studies examine earlier time periods, starting between 15 to 30 years before the time series assessed here. We speculate that this may indicate a shifting baseline effect, whereby the ground beetle communities investigated here may have been altered prior to the start of our observation period, with only taxa robust against anthropogenic disturbances remaining.

In partial support of Hypothesis 2, taxonomic turnover (SERa) increased with temperature and tended to increase with precipitation across all sites. Regionally, we found an increasing species exchange rate in the SPW region, while in the CHO region, species disappearance decreased and species appearance increased over time. These results illustrate that long-term ecological trends can vary across spatial scales^99^. Although research focusing on single^50,98^ or few sites^40,47^ are indispensable to understand the influence of local scale drivers, our results illustrate that localized changes in diversity may not hold at broader spatial scales^97^.

Precipitation was the most important environmental driver of site-based trends in ground beetle communities. Activity density of habitat specialists increased with increasing precipitation while Simpson’s diversity, evenness (EVE), and functional dispersion, all declined. This suggests that habitat specialists became the dominant ground beetle group in moist conditions, at the expense of habitat generalists. High rainfall increases ground beetle habitat specialization, a consequence of specific adaptations to moist conditions^56,57^. Site-based trends in the species exchange rate (SERa) increased with site-based trends in temperature and declined with land use intensities. Consequently, variable environmental conditions at the investigated sites may increase selection for species that are better adapted to more extreme living conditions^40–42,52^. Accordingly, high land use intensities might lower the exchange rate of species. However, our meta-analytic approach, while necessary for a standardized assessment of all examined responses besides activity densities, collapses climate and ground beetle response values from each site into one overall trend, and thus has reduced power to detect the influence of environmental drivers, especially if sites have high variability across years.

Considering temporal variation in addition to spatial differences revealed climate-driven influences on the overall activity density of all ground beetles and on activity densities of individual functional groups that were not evident from our site-based meta-analytic approach. High activity densities at intermediate precipitation suggests moderate rainfall either provided more resources for ground beetles or increased ground beetle movement relative to extreme rainfall conditions, in turn increasing trap catch. While temperature did not have any influence on the overall activity density, three functional groups (dimorphic beetles, imago hibernators, larval hibernators) tended to decline with increases in annual temperature. This suggests decreasing overwintering survival as a consequence of desiccation^50^ and a decline of beetles with reduced abilities to relocate to habitats that are within their temperature tolerance range. Microhabitat conditions and local habitat structure can also drive ground beetle activities at smaller spatial scales^36,100^. The sampling sites assessed in our study covered many heterogeneous habitat types and corresponding microclimatic conditions. Disentangling how interactions between these conditions contributed to the observed and unobserved influences of climate on activity density of functional groups is a fruitful avenue for future work.

We did not find support for our third hypothesis that LUI would have a linear negative effect on temporal trends in taxonomic and functional diversity. However, across an increasing gradient of LUI, ground beetles varied non-linearly both in activity density and by functional groups. Activity densities of all functional groups had unimodal responses to LUIs. Ground beetle taxonomic reorganization (SERa) was highest at low LUIs, potentially due to a larger species pool. At intermediate LUIs, specialists, winged beetles, and imago-hibernators proportionally dominated the ground beetle communities, while very high land use intensities reduced activity densities of ground beetles independently of their functional traits.

Our study is subject to several common challenges in long-term research that may limit our ability to capture trends in the sampled ground beetle communities. While all but one of our 40 sites cover periods of at least 10 years (average 13.9 years), a caveat of our temporal analysis is the comparably lower number of sampling years ranging from 4 to 10 (averaging 7 years). This particularly applies to the Elbe region, which had the fewest sampling years. However, regional trends in the Elbe region were similar to those from the other four regions, indicating that overall, our analyses reflect well the temporal patterns in the ground beetle communities across the last two decades. Additionally, the peak of human pressures may have been reached before the onset of our observation period^24^ starting in 1999. The sampled community was potentially already at the “bottom of the barrel”—that is major long-term changes in ground beetle communities prior to 1999 may have filtered out particular taxa resulting in no evidence of recent trends^52,101^.

In comparison to previous long-term studies on ground beetle communities^40,41,98^, a major strength of our study is its high number of sites from multiple regions. While heterogeneous habitat types covering different local environmental conditions might result in averaged-out trends at larger scales, similar patterns across all five regions suggest rather low temporal variation in the assessed ground beetle communities. However, and importantly, responses of functional group activity densities had several unimodal or u-shaped responses to climate and land use. Such non-linear responses challenge trend detection, especially when sample number is limiting. Additionally, incorporating both local scale microclimate and habitat structure poses a challenge in long-term and large scale-studies of invertebrates, but remains a key consideration for future work. Finally, we echo recent calls for distributed and standardized long-term monitoring schemes to unravel temporal changes in biotic communities and their driving forces^102^.

## Conclusions

Ground beetle activity densities peaked at intermediate rates of annual precipitation and intermediate land use intensities. Hump-shaped responses may either result from intermediate conditions having the greatest overlap with most species habitat needs, or be indicative of optimal environmental conditions for ground beetles. While we detected no overall temporal trends of ground beetle communities, this result should be interpreted with caution. Temporal changes in a few community metrics in certain regions emphasize the need for more large-scale and long-term monitoring schemes to understand the role of spatial drivers. Our study further outlines the need to consider functional diversity measures in addition to more traditional taxonomic metrics to better understand the complexity of spatiotemporal changes in biotic communities.

## Supplementary Information

Supplementary Information.
