# Supplementary material for: Long-term data reveal unimodal responses of ground beetle abundance to precipitation and land use but no changes in taxonomic and functional diversity
Source: Sci Rep. 2021 Sep 1;11:17468. doi: 10.1038/s41598-021-96910-7 (PMC8410911; doi:10.1038/s41598-021-96910-7)
Supplement: Supplementary file 1 — Supplementary Information. [file 41598_2021_96910_MOESM1_ESM.pdf]

Supplementary materials

Title

Long-term data reveal unimodal responses of ground beetle abundance to precipitation and land use but no changes in taxonomic and functional diversity

Authors

Petr Zajicek[1*], Ellen A. R. Welti[1], Nathan J. Baker[1], Kathrin Januschke[2], Oliver Brauner[3], Peter Haase[1,4]

[1]Senckenberg Research Institute and Natural History Museum Frankfurt, Department of River Ecology and Conservation, Gelnhausen, Germany

[2]University of Duisburg-Essen, Department of Aquatic Ecology, Essen, Germany

[3]Office for Zoology, Vegetation and Conservation (Büro für Zoologie, Vegetation und Naturschutz), Eberswalde, Germany

[4]University of Duisburg-Essen, Faculty of Biology, Essen, Germany

*corresponding author: pzajicek2008@gmail.com

Contents

Supplementary methods

Supplementary tables A1 - A3

Supplementary figures A1 – A12

Supplementary methods

Approach one: Overall and regional temporal trends and the influence of drivers

To assess temporal trends in community metrics and climate variables overall and within each region, we applied a two-step procedure consisting of site-specific time-series based trend calculations and a subsequent meta-analytical approach[1]. We first calculated effect sizes of temporal trends of each community metric and climatic variable for each site and its observation period (= within-site comparison)[1]. For this purpose, we applied the Mann-Kendall trend test and derived its S-statistic and variance as measures of trend effect sizes[2,3]. We accounted for temporal autocorrelation when indicated in auto- and cross-covariance and correlation functions[4,5] and applied a modified Mann-Kendall trend test for autocorrelated data[6] when temporal autocorrelation was detected. Next, we used meta-analysis mixed-effects models (MAMEM), which use effect sizes derived in the Mann-Kendall trend tests[1,7,8], to test 1) for trends in community metrics overall and within regions, 2) for overall trends in temperature and precipitation and 3) how trends in temperature and precipitation influence, together with site-specific LUI's, trends in community metrics. Site is our replicate level for all statistical analyses of overall and regional trends and the drivers of overall trends. We also included study length as an additional fixed effect to account for variable observation periods between sites. A spatial autocorrelation structure was added as random effect[8] to all models and was nested within region for overall models to also account for between region heterogeneities, particularly a potential seasonal effect as one region (RMO) was sampled in a different season. In total, we fitted one model for each response metric and each of the above scenarios

resulting in 65 MAMEM. In each MAMEM, we accounted for spatial autocorrelation according to Maire et al. 2019[9]. The Land Use Index was square-root transformed, observation period was log-transformed, and all predictors included in each MAMEM were standardized to a mean of zero and a standard deviation of one. We report model coefficients as the trend mean effect size (TMES) and standard error (SE) following Maire et al. 2019[9] for the overall trends in community metrics and in climate variables and for trends within regions. All predictors included in the MAMEM models showed little collinearity (Pearson's $|r| < 0.6$). To identify the most important drivers of trends in community metrics, we applied an information-theoretic approach to model selection and multi-model inference using the corrected Akaike's information criterion (AICc) [10,11] to assess the model fit. We retained only models with Δ AICc ≤ 2 to the model with the lowest AIC_c[10]. We report the z-values as effect sizes of the identified drivers of temporal trends in community metrics.

Approach two: Effects of spatio-temporal variation in drivers on activity density and functional groups

To examine the effects of annual temperature and precipitation within each sampling year and site-specific LUI on activity density and functional groups, we used gls models. All gls models included an autoregressive term to account for temporal autocorrelation and a grouping factor of site nested within region to account for repeated sampling within sites, region-specific sampling methodology, and site-specific habitat heterogeneity. We first ran gls models including second order polynomials of the three fixed driver variables (temperature, precipitation and LUI_1000) to account for intermediate effects. When second order polynomials were not significant, we removed them from reported gls models. Activity density,

temperature and precipitation were log10 transformed, and LUI_1000 was square root transformed to meet normality and heteroscedasticity assumptions. We calculated one gls model predicting activity density and seven gls models predicting functional groups. Additionally, we wished to examine how climate and land use influenced the dominance of functional groups. As all functional group abundances were correlated, we first calculated the percent individuals of each functional group and then conducted a Principal Component Analysis (PCA) on the percent values (Appendix Fig. A1). Functional group percent values were correlated with either the first or second principal components (Appendix Table A1). We then ran two additional gls models using the first and second principal components (PC1 and PC2) as response variables. In total, we fitted ten gls models.

References

1. Pilotto, F. *et al.* Meta-analysis of multidecadal biodiversity trends in Europe. *Nature Communications* **11**, 3486 (2020).
2. Kendall, M. G. *Rank correlation methods*. (Griffin, 1948).
3. Mann, H. B. Nonparametric tests against trend. *Econometrica* **13**, 245–259 (1945).
4. Dornelas, M. *et al.* Quantifying temporal change in biodiversity: challenges and opportunities. *Proceedings of the Royal Society B: Biological Sciences* **280**, 20121931 (2013).
5. Venables, W. N. & Ripley, B. D. *Modern applied statistics with S*. (Springer-Verlag, 2002). doi:10.1007/978-0-387-21706-2.
6. Hamed, K. H. & Rao, R. A. A modified Mann-Kendall trend test for autocorrelated data. *Journal of Hydrology* **204**, 182–196 (1998).

7. Daufresne, M., Lengfellner, K. & Sommer, U. Global warming benefits the small in aquatic ecosystems. *PNAS* **106**, 12788–12793 (2009).

8. Viechtbauer, W. Conducting meta-analyses in R with the metafor package. *Journal of Statistical Software* **36**, 1–48 (2010).

9. Maire, A., Thierry, E., Viechtbauer, W. & Daufresne, M. Poleward shift in large-river fish communities detected with a novel meta-analysis framework. *Freshwater Biology* **64**, 1143–1156 (2019).

10. Anderson, D. R. & Burnham, K. P. Avoiding pitfalls when using information-theoretic methods. *The Journal of Wildlife Management* **66**, 912–918 (2002).

11. Burnham, K. P. & Anderson, D. R. Multimodel inference: understanding AIC and BIC in model selection. *Sociological Methods & Research* **33**, 261–304 (2004).

Supplementary Tables

Table A1. Correlations between PCA scores and percent dominance of functional groups for the PCA shown in Fig. A3. Functional groups included generalists (GEN), predators (PRE), dimorphic (DIM), winged (WIN), larval hibernators (LAR), and imago-hibernators (IMA). Negative values are shaded in red, positive values are shaded blue, and colors are shaded darker with increasing correlation strength.

	PC1	PC2
GEN	-0.80	0.19
PRE	-0.12	-0.74
IMA	-0.72	-0.06
LAR	0.37	0.64
WIN	-0.92	0.15
DIM	0.85	-0.13

Table A2. Drivers of activity density of ground beetle functional groups using generalized least squares models for generalists (A), specialists (B), predators (C), winged beetles (D), dimorphic beetles (E), larval hibernators (F), and imago-hibernators (G). All models included an autoregression term to account for temporal autocorrelation, a grouping factor of site nested within region to account for repeated sampling within sites. The second order polynomial terms were included when significant. Driver estimates are shown in bold when significant. Significant responses of functional group activity densities to LUI and climate are shown in Fig. A10 and Fig. A11 respectively.

	**Est.**	**SE**	**t-value**	***P***
**A.)** Generalist Activity Density				
Intercept	-0.168	1.605	-0.105	0.917
Temperature	-0.473	0.702	-0.674	0.501
Precipitation	0.868	0.913	0.950	0.343
Precipitation, 2nd poly	**-2.159**	**1.004**	-2.150	0.032
LUI_1000m	**-5.120**	**1.432**	-3.575	<0.001
LUI_1000m, 2nd poly	**-8.668**	**1.380**	-6.283	<0.001
**B.)** Specialist Activity Density				
Intercept	8.008	4.443	1.802	0.073
Temperature	-1.950	1.337	-1.458	0.146
Precipitation	-0.862	0.687	-1.254	0.211
LUI_1000m	**-6.734**	2.755	-2.444	0.015
LUI_1000m, 2nd poly	**-15.926**	2.657	-5.995	<0.001
**C.)** Predator Activity Density				
Intercept	-4.281	1.936	-2.211	0.028
Temperature	-1.111	0.644	-1.724	0.086
Precipitation	**0.964**	0.293	3.291	0.001
LUI_1000m	-3.060	1.766	-1.733	0.084
LUI_1000m, 2nd poly	**-9.970**	1.699	-5.868	<0.001

	**Est.**	**SE**	**t-value**	***P***
**D.)** Winged Beetle Activity Density				
Intercept	2.801	2.863	0.979	0.329
Temperature	-1.978	1.252	-1.580	0.115
Precipitation	-0.330	1.629	-0.203	0.840
Precipitation, 2nd poly	**-3.940**	1.791	-2.200	0.029
LUI_1000m	-2.795	2.554	-1.095	0.275
LUI_1000m, 2nd poly	**-13.418**	2.460	-5.454	<0.001
**E.)** Dimorphic Beetle Activity Density				
Intercept	0.358	2.941	0.122	0.903
Temperature	0.551	1.456	0.379	0.705
Temperature, 2nd poly	**3.294**	1.286	2.561	0.011
Precipitation	-0.295	0.461	-0.641	0.522
LUI_1000m	**-6.403**	1.721	-3.721	<0.001
LUI_1000m, 2nd poly	**-8.766**	1.657	-5.290	<0.001
**F.)** Larval-hibernator Activity Density				
Intercept	5.565	5.520	1.008	0.314
Temperature	1.353	2.743	0.493	0.622
Temperature, 2nd poly	**6.625**	2.420	2.738	0.007
Precipitation	-1.504	0.865	-1.739	0.083
LUI_1000m	**-10.939**	3.265	-3.351	<0.001
LUI_1000m, 2nd poly	**-8.477**	3.143	-2.697	0.007
**G.)** Imago-hibernator Activity Density				
Intercept	1.047	3.544	0.295	0.768
Temperature	-1.162	1.823	-0.637	0.525
Temperature, 2nd poly	**3.185**	1.586	2.009	0.046
Precipitation	-0.402	0.555	-0.725	0.469
LUI_1000m	**-5.292**	2.309	-2.292	0.023
LUI_1000m, 2nd poly	**-14.151**	2.221	-6.373	<0.001

Table A3. Drivers of the principal component (PC) scores representing dominance of functional groups. The Prinicipal Component Analysis is visualized in Fig. A1 and correlations with percent of total (dominance) of functional groups are provided in Table A1. High values of the first PC suggest dominance of dimorphic beetles, while low values suggest dominance of specialists, winged beetles and those overwintering as imagines. High values of the second principal component suggest dominace of beetles overwintering as larva while low values suggest dominance of predators. The negative correlation of precipation with PC2 thus suggests high precipitation favors dominance of predators and reduces dominance of larval-hibernators (see Fig. A12 for visualization). No second order polynomials of drivers predicted PC dominance scores.

	**Est.**	**SE**	**t-value**	***P***
**A.)** PC1 (+ %DIM, - %SPE, - %WIN, - %IMA)				
Intercept	-5.852	3.001	-1.950	0.052
Temperature	1.370	0.991	1.383	0.168
Precipitation	0.389	0.455	0.856	0.393
LUI_1000m	-1.103	2.628	-0.420	0.675
LUI_1000m, 2nd poly	**7.486**	2.529	2.961	0.003
**B.)** PC2 (+ %LAR, - %PRE)				
Intercept	5.283	2.094	2.523	0.012
Temperature	0.348	0.636	0.547	0.585
Precipitation	**-0.910**	0.327	-2.782	0.006
LUI_1000m, 2nd poly	-0.014	0.021	-0.661	0.509

Supplementary Figures

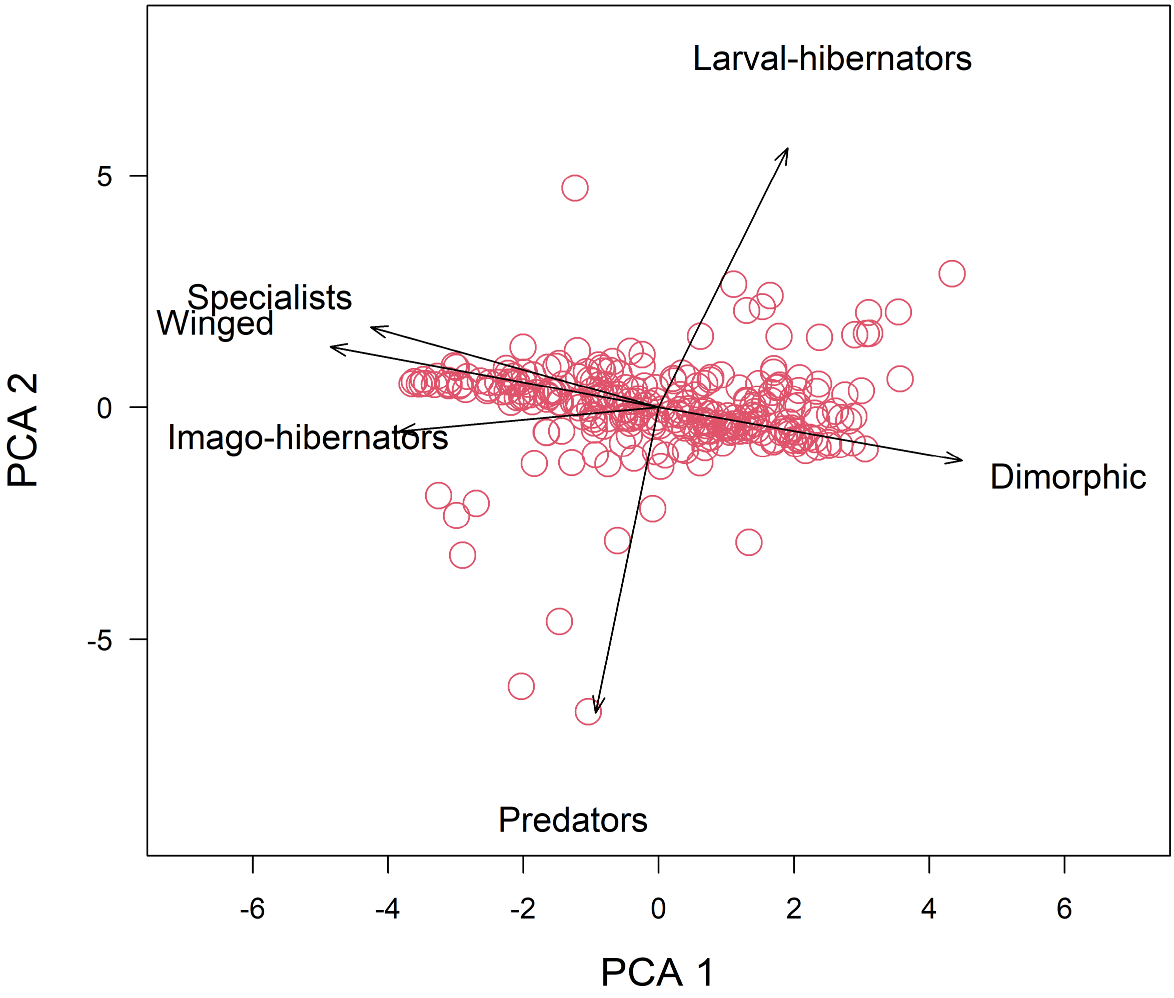

Figure A1. Principal component analysis of the percent of total of the functional groups: generalists, predators, dimorphic, winged, larval hibernators, and imago-hibernators. Generalists were not included in the PCA as the sum of percent of generalists and specialist was always 100.

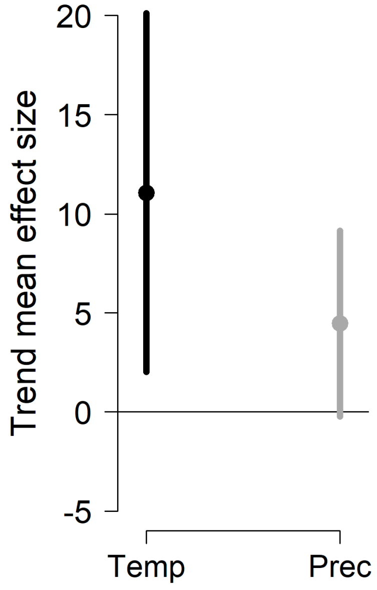

Figure A2. Overall changes in temperature (Temp) and precipitation (Prec) over time across the 40 sites. Lines show 95% confidence intervals with black color indicating a significant trend and a grey colored line indicating a non-significant trend.

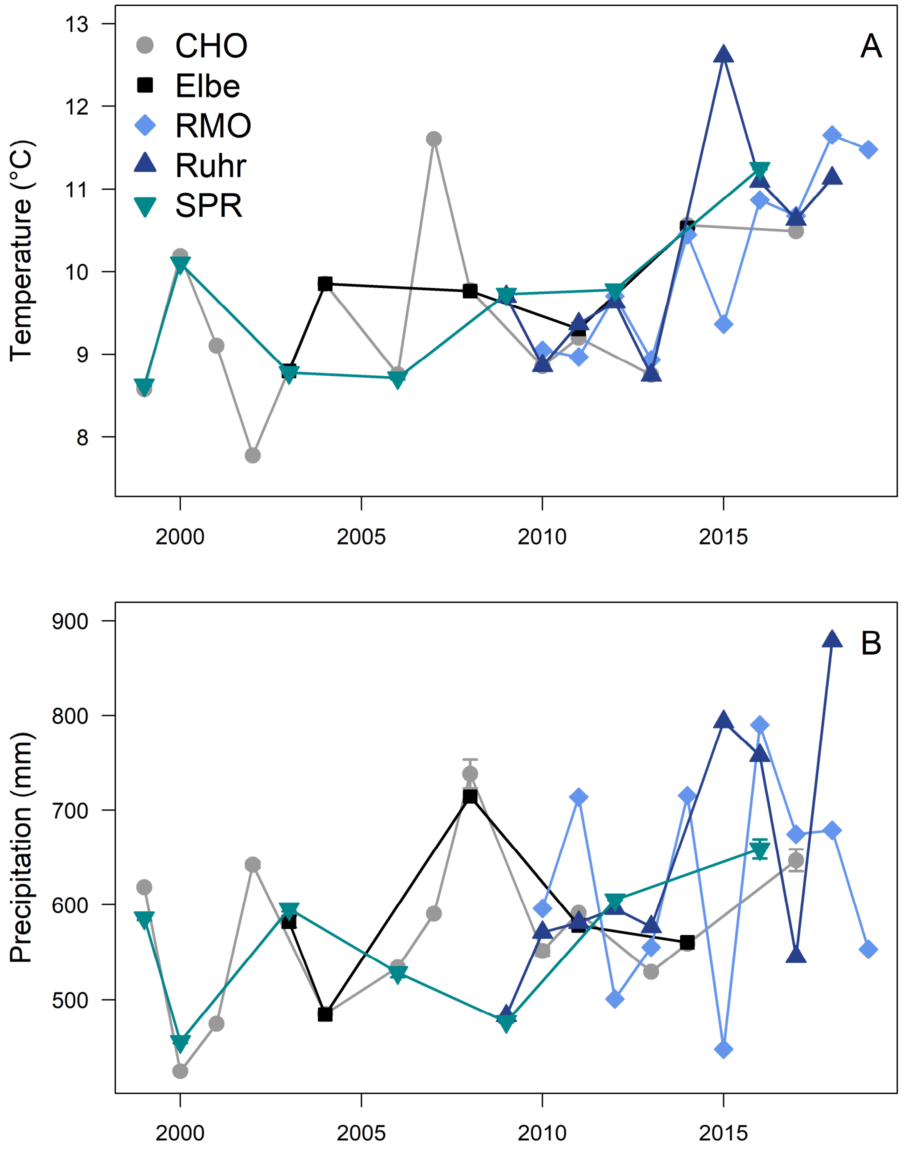

Figure A3. Regional changes in temperature (A) and precipitation (B) across the five regions and study period. Average temperature and cumulative precipitation are derived at each site and year from the 12 months prior to sampling. Each point represents the average value of the sites within each region. Error bars represent one standard error of site averages but are not always visible as sites within regions had very similar climate conditions.

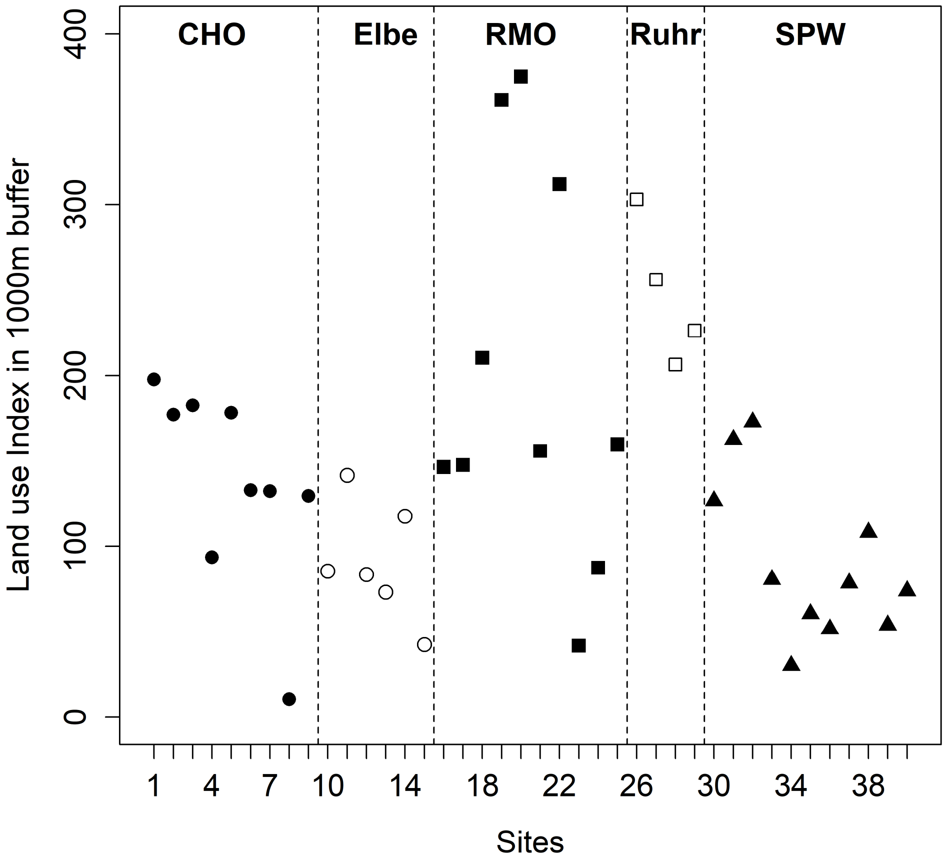

Figure A4. Land use intensities in 1000m buffers around sampling sites within the five investigated regions.

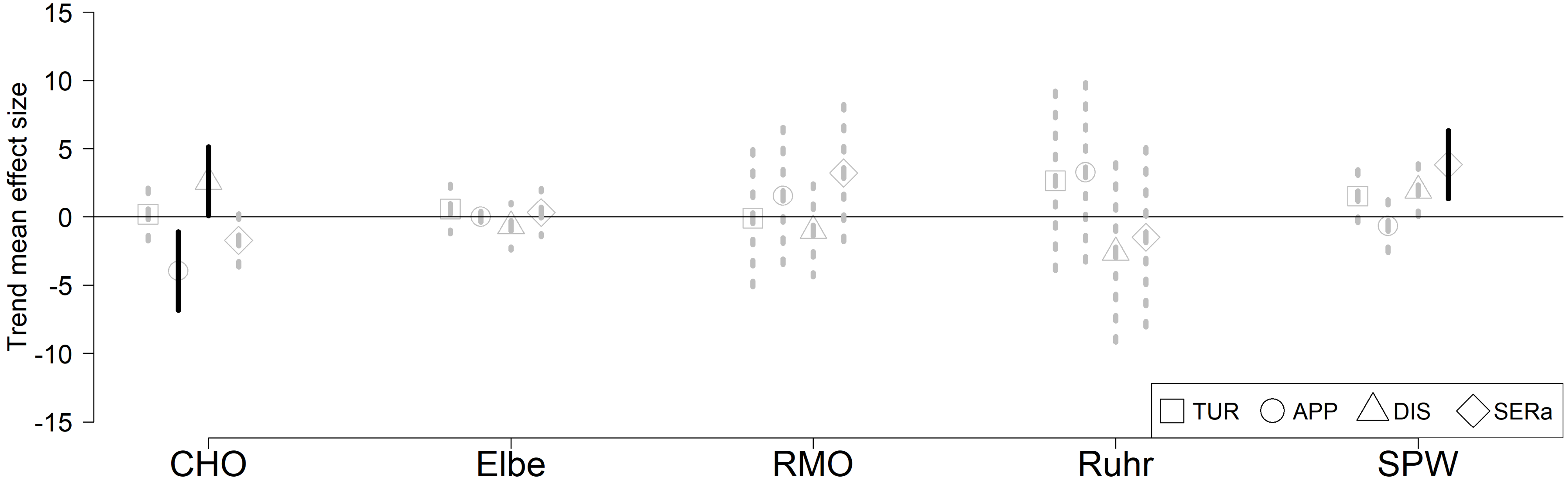

Figure A5. Regional trends in the four turnover metrics (descriptions of abbreviations are provided in Table 1). Vertical lines represent 95% confidence intervals with black lines indicating significant trends and with dashed grey lines indicating non-significant trends.

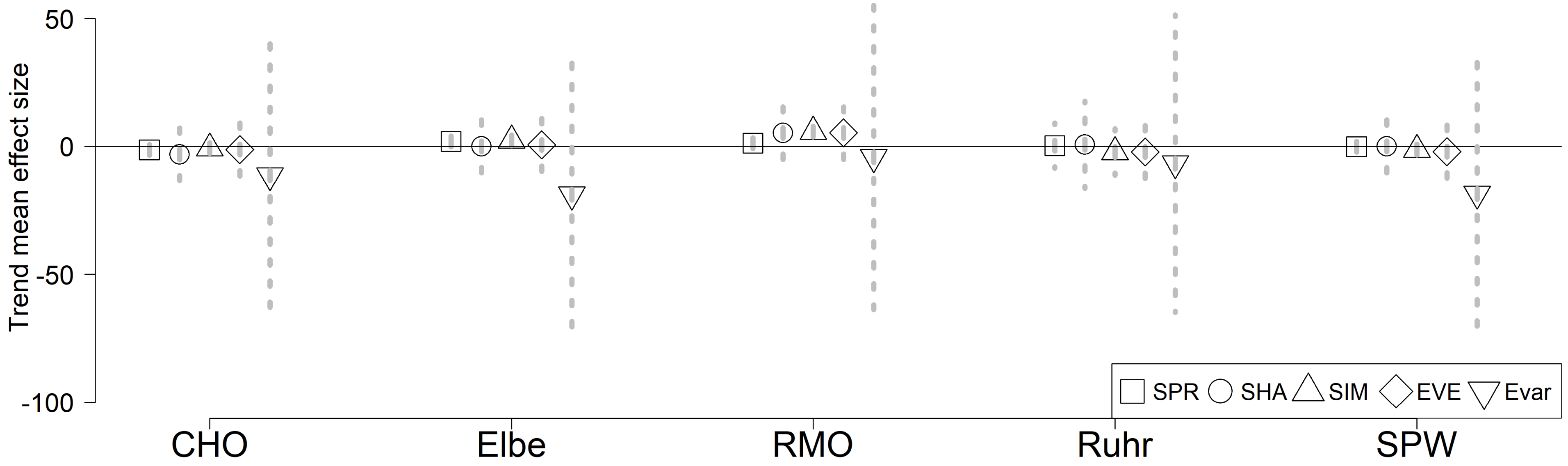

Figure A6. Regional trends in community metrics relating to taxonomic diversity that were not significant in any region (descriptions of abbreviations are provided in Table 1). Lines represent 95% confidence intervals. While the negative estimates of Evar with high confidence intervals within regions are contradictory to the overall trend; however, this is caused by the spatial autocorrelation structure accounted for in the statistical analyses (MAMEM).

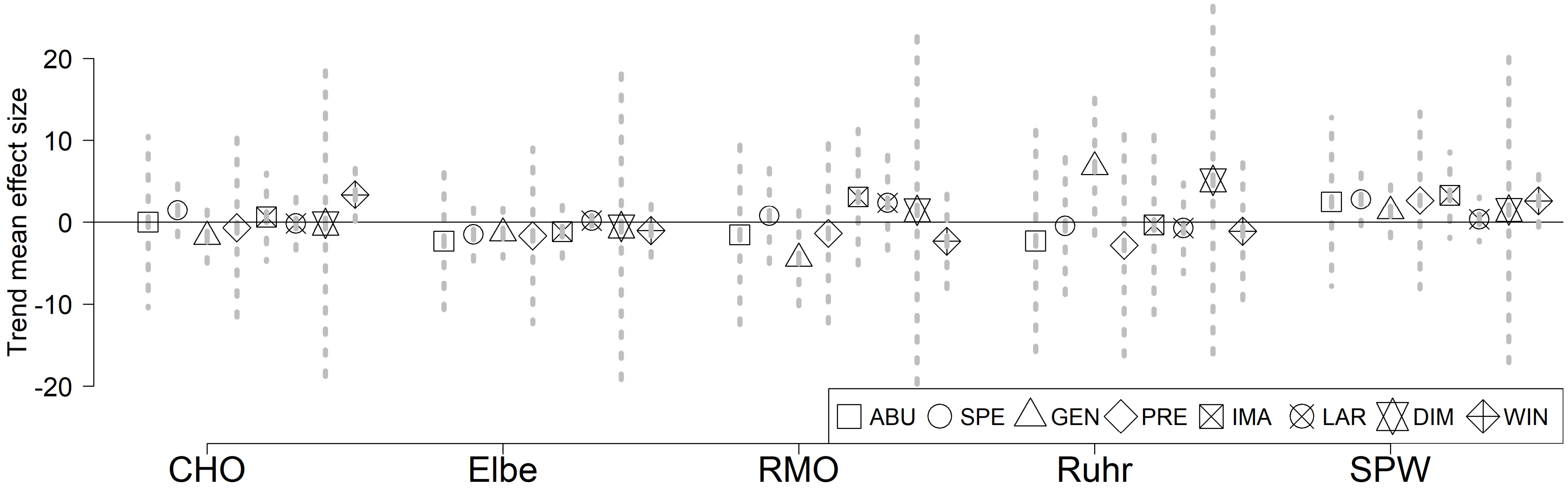

Figure A7. Regional trends in community metrics relating to activity density (ABU) and activity densities of functional groups that were not significant in any region (descriptions of abbreviations are provided in Table 1). Lines represent 95% confidence intervals.

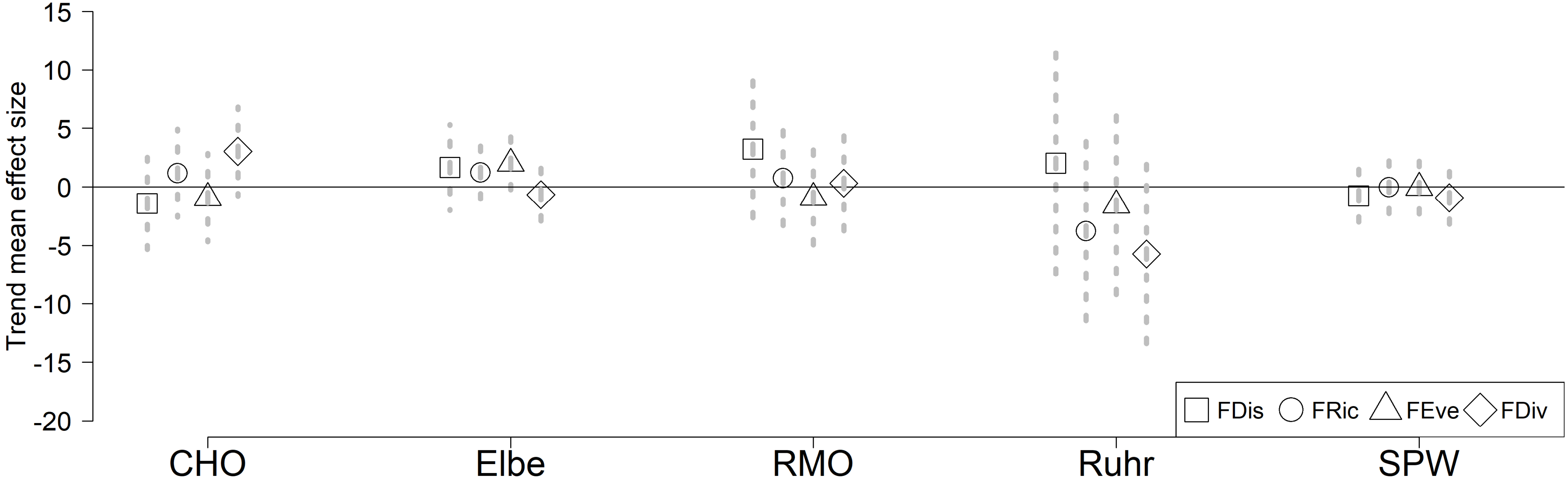

Figure A8. Regional trends in community metrics relating to functional diversity that were not significant in any region (descriptions of abbreviations are provided in Table 1). Lines represent 95% confidence intervals.

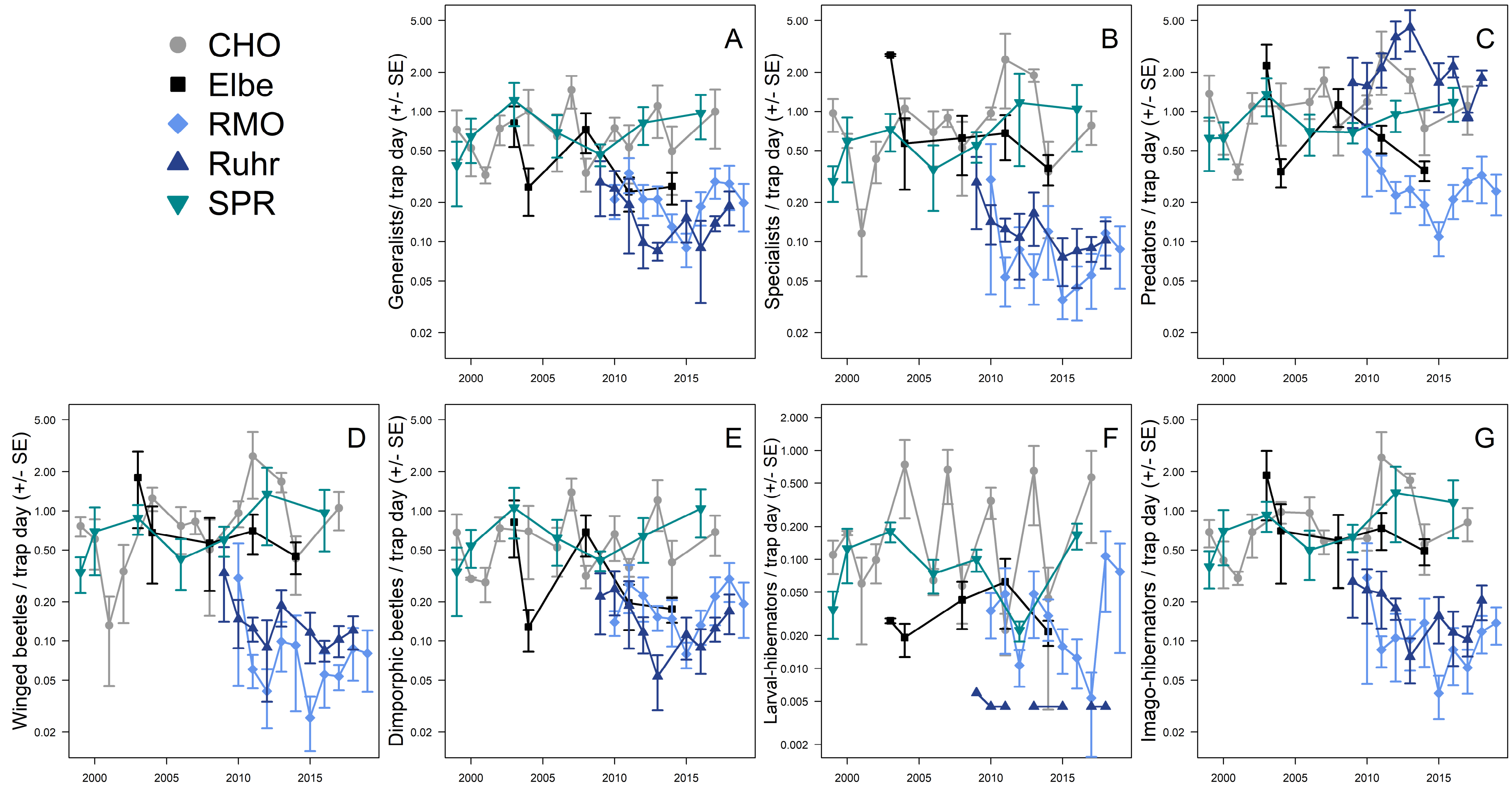

Figure A9. Temporal changes in activity density for the five regions of the seven functional groups of generalists (A), specialists (B), predators (C), winged beetles (D), dimorphic beetles (E), larval hibernators (F), and imago-hibernators (G). Error bars represent 1 standard error calculated from the values within each of the sites within regions with sampling within each year. Y-axes are identical for all panels except for larval hibernators (F), which had much lower activity densities than other function groups.

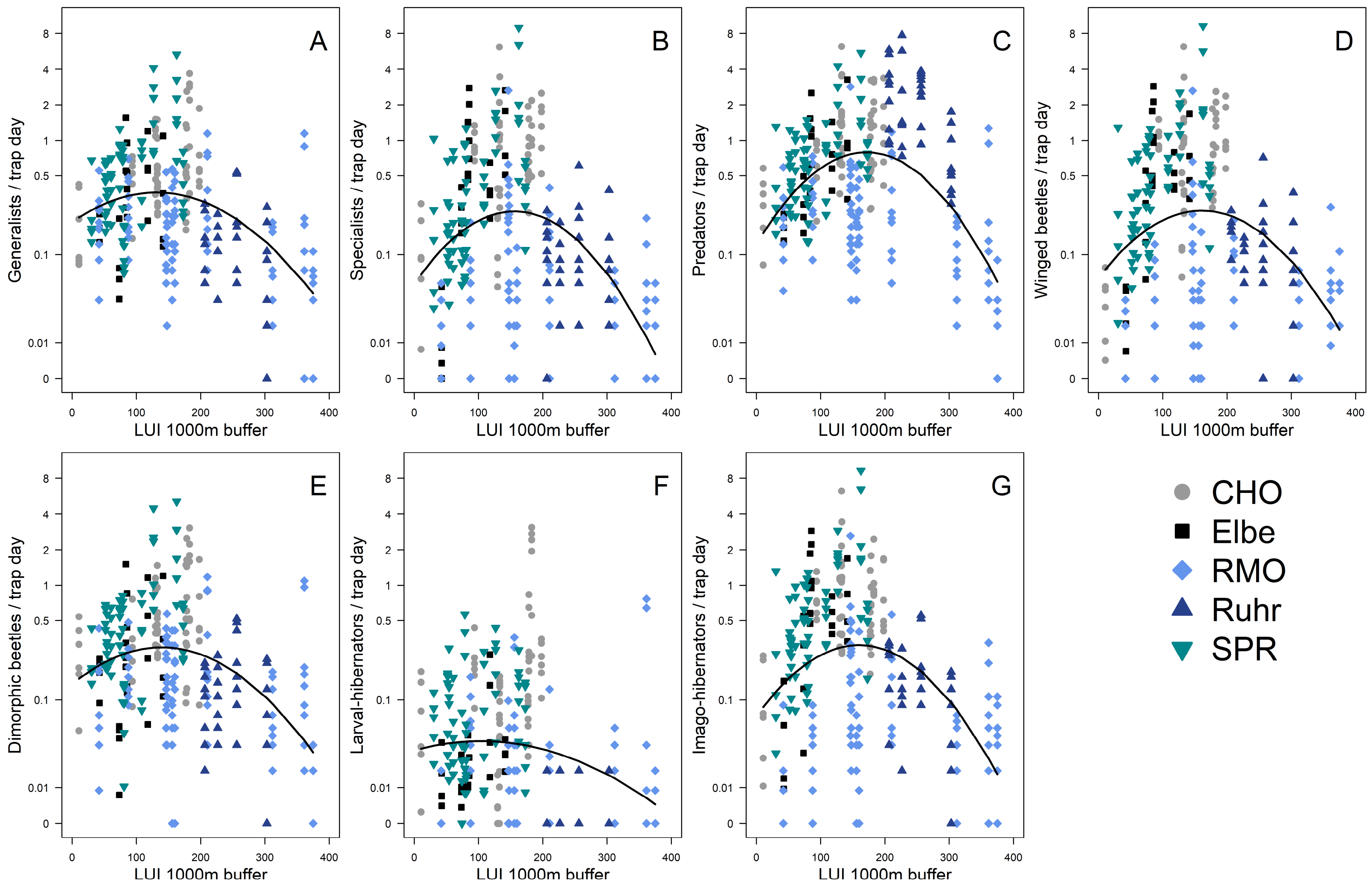

Figure A10. Responses of functional group activity densities to land use intensities. All functional groups (A-G) had unimodal responses (significantly negative polynomial terms) to land use intensities (Table A2). Each point represents the activity density of the corresponding functional group within a site and year.

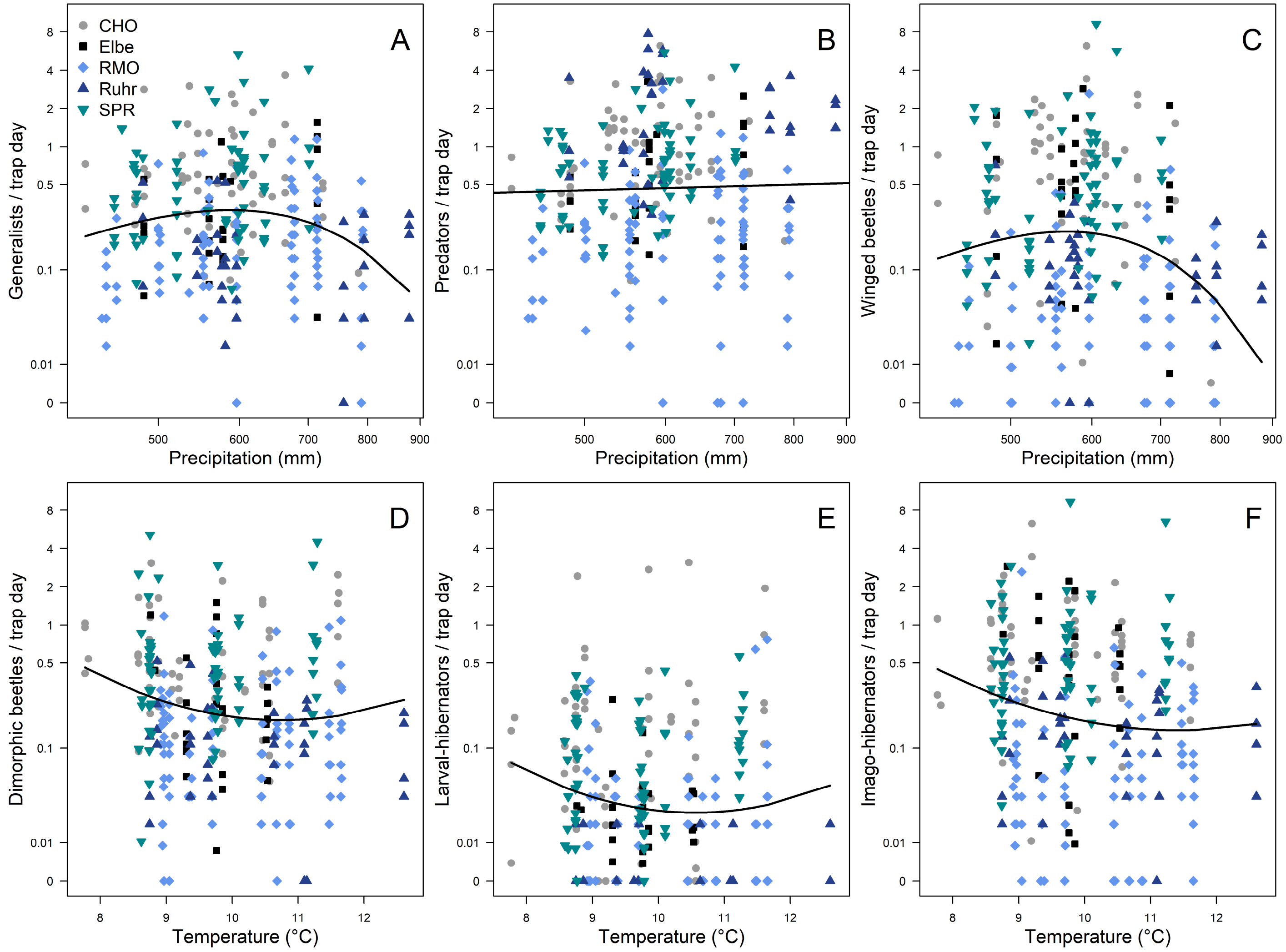

Figure A11. Responses of functional group activity densities to precipitation and temperature when significant in GLS models (Table A2). Generalists (A) and winged-beetles (C) showed a unimodal response (significantly negative polynomial term) to precipitation, predators increased with precipitation (B), and dimorphic beetles (D), larval-hibernators (E) and imago-hibernators (F) showed u-shaped responses (significantly positive polynomial term) to temperature. Each point represents the activity density of the corresponding functional group within a site and year.

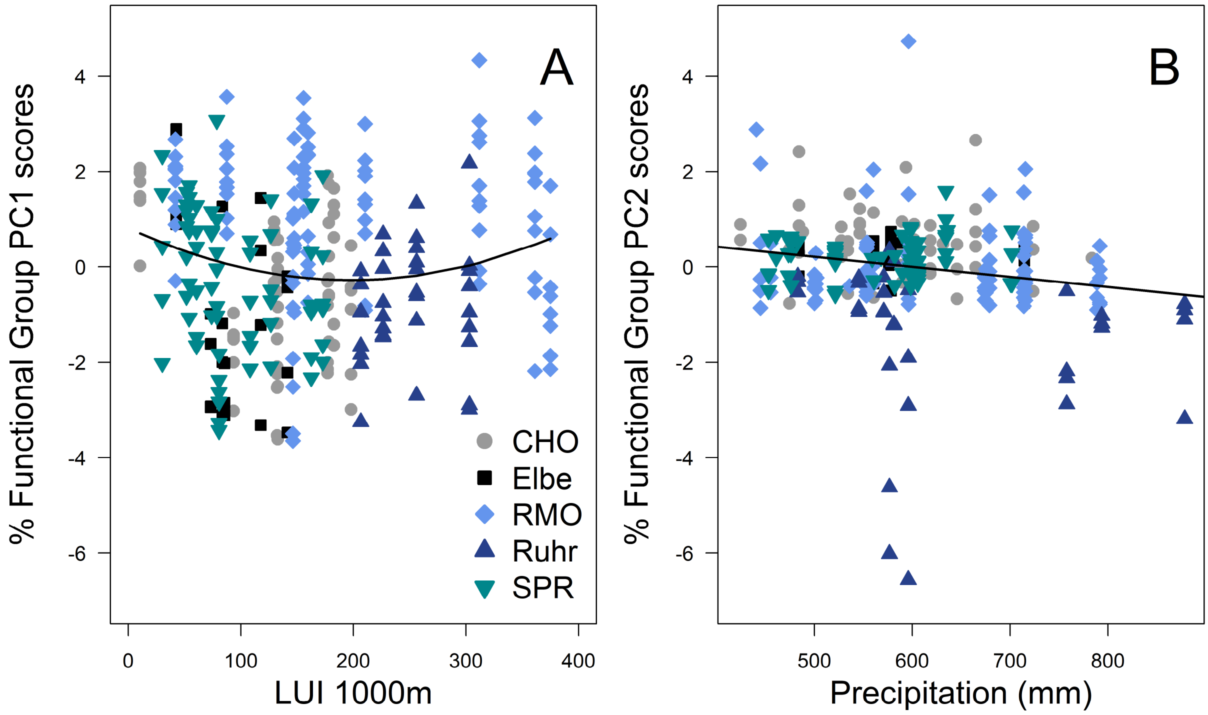

Figure A12. Response to land use (A) and precipitation (B) of the principal component axes of the Principal Component Analysis (PCA) of functional group percent dominances. See Fig A1 for PCA visualization, Table A1 for correlations between PC scores and percent ground beetle functional groups, and Table A3 for results of models of PC score responses to climate and land use intensity drivers.PC1 is negatively correlated to percent habitat specialist, imago-hibernators and winged beetles and positively correlated with percent dimorphic beetles. PC2 is negatively correlated with percent predators and positively correlated with percent larval-hibernators.
